# Supplementary material for: GeNNet: an integrated platform for unifying scientific workflows and graph databases for transcriptome data analysis
Source: PeerJ. 2017 Jul 5;5:e3509. doi: 10.7717/peerj.3509 (PMC5501156; doi:10.7717/peerj.3509)
Supplement: Supplemental Information 1 [file peerj-05-3509-s001.pdf]

GeNNet: An Integrated platform for unifying scientific workflow management and graph databases for transcriptome data analysis

Raquel L. Costa and *Luiz Gadelha* and *Marcelo Ribeiro-Alves* and *Fabio Porto*

National Laboratory for Scientific Computing (LNCC), National Laboratory of Cancer (INCA), National Institute of Infectology, Oswaldo Cruz Foundation (Fiocruz)

e-mail: quelopes@gmail.com

References

GeNNet: An Integrated Platform for Unifying Scientific Workflow Management and Graph Databases for Transcriptome Data Analysis. DOI: https://doi.org/10.1101/095257

Table of Contents

- Run the GeNNet
- Licence
- About
  - A: A user-friendly Shiny-based web interface to the workflow experiment
  - B: Access RStudio
  - C: Access the Graph Database

Run GeNNet

0 - Install Docker

For Windows: link

For Mac: link

For Linux distribution (ex: Ubuntu) link

1 - Pull our Docker image from Dockerhub.

See in: link

```
$ docker pull quelopes/gennet
```

2 - Run the Docker image

```
$ docker run -d -p 8787:8787 -p 3838:3838 -p 7474:7474 -v dir/Data:/home/rstudio/Data -v dir/Results/:/I
```

Obs: The ports must be available/free on the hosting system.

Licence

GNU GENERAL PUBLIC LICENSE version 3 by Free Software Foundation, Inc. Read the original GPL v3.

About

GeNNet is a platform to execute experiments using transcriptome data, specially developed for analyzing microarray platforms currently available for human, rhesus, mice and rat. The structure is composed by three different parts: **A** – A user-friendly Shiny-based web interface to the workflow experiment; **B** – Executing or editing the workflow experiment using RStudio (for advanced users and developers) and **C** – Accessing the resulting gene interaction graph database using Neo4j. Figure 1 (below) describes the GeNNet architecture.

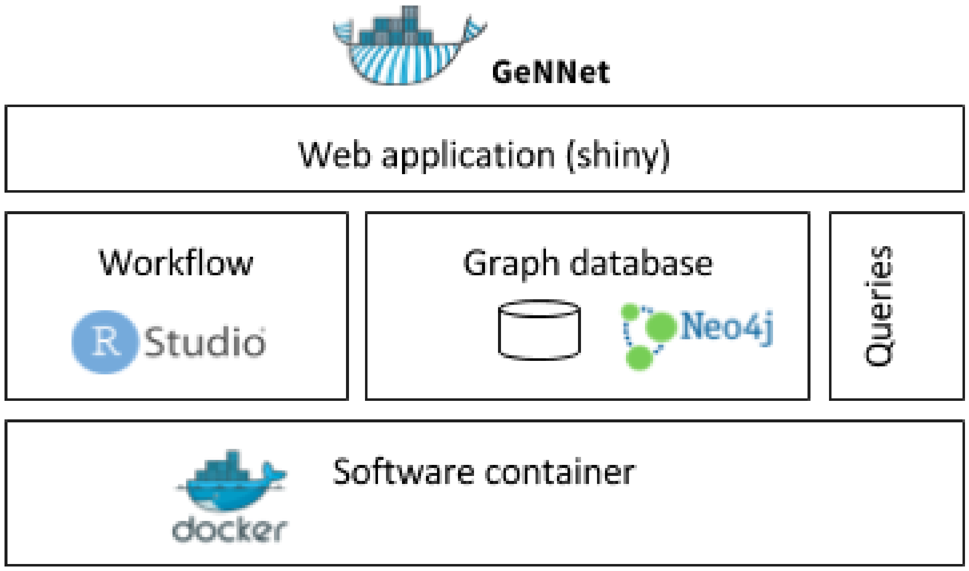

Figure 1: Figura 1: Framework of GeNNet.

ARCHIVES

Before starting the `gennet` software container in Docker the user needs to upload the raw data and pheno data description. To do so, the user needs to create a directory with the name of the experiment (for instance `GSE62232`) and create two subdirectories within this directory. The first directory should be named `Data` and the pheno data description should be copied to it as comma or tab-separated values. The second subdirectory should be named `Results`, which will receive the files generated by the workflow. Within the `Data` subdirectory, another subdirectory called `CEL` should be created and all the raw data that will be analyzed should be copied to it. More details about how to prepare this archive are described bellow.

Upload Phenodata

The pheno data is loaded when starting the GeNNet container. However, users can upload their own pheno data matrices. Separator can be choosen to be tab (\t) or comma (,). The current matrix can be visualized in the `PhenoData` tab.

The input file must be structured using mainly two columns: a column named `SETS` for the experimental design, and a column named `SAMPLE_NAME` for the names of the files containing raw sample expression matrix data.

CEL archives

The CEL file archives are loaded when the container GeNNet is started. They contain raw data from Affymetrix microarray platforms. You can see the data uploaded accessing the tab `CEL Archives Data`.

A – A user-friendly Shiny-based web interface to the workflow experiment

A Shiny-based web interface was developed to provide a user-friendly way to execute the workflow described above. We developed an easy-to-use layout for providing the parameters and automatically executing all steps of the workflow experiment.

*** Open your browser***

`localhost:3838/gennet`

Experiment information

This panel contains some fields for writing information about the experiment which includes `Experiment name` and `Overall design`. We recomend using a short string in `Experiment name`. For instance GSE62232 (accession number of GEO link) or other short denomination. Observation, the experiment name should be the same of the pheno data file prefix (e.g. if the pheno data filename is GSE62232.csv, the experiment name should be GSE62232). In `overall design` users can describe information about their experiment or copy the descriptive tag from GEO.

Normalization Parameters

`Normalization parameters` allows users to choose the method that will be applied for normalization. Currently, GeNNet contains two methods available, `mas5` and `rma`. For more details about these methods one can access the `affy` package manual in Bioconductor in link.

Set Parameters to apply Differential Expression

`Differential expression (DE)` inference analysis allows for the recognition of groups of genes modulated (up- or down-regulated) in a biological system when compared among one or more experimental conditions. In many situation this is a core step of the analysis and there are a great diversity of experimental designs (such as control versus treatment, consecutive time points, etc) allowing the inference. In our platform, we use the `limma` package to select the DE genes on single-factor experimental designs based on a gene-based hypothesis testing statistic followed by a correction of multiple testing given by the `False Discovery Rate (FDR)`. Furthermore, a subset of DE genes can be selected based on a up- and down-regulation, expressed as a logarithmic (base 2) fold-change (logFC) threshold.

The initial cut-off values (log2(Fold-Change) > 1 and FDR < 0.05) chosen are the most used and recommended in the literature but the cut-off values can be adjusted. Results of this step are displayed as Volcano plots and Matrices containing the DE genes.

Platform parameters

The platform parameters consist of choosing the `platform` and choosing the `organism`. The `platform` consists of the annotation specifically developed for the microarray platform. Currently GeNNet contains four platforms with the corresponding number associated in the `GEO` repository. The `organism` is related with the organism of annotation in which the microarray platform was based (Human, rhesus, mice and rat) are included.

Clusterization Parameters

This step consists in analyzing which aggregated genes have a similar pattern (or level) of expression. We incorporated clusterization analysis including hierarchical methods, k-medoids from the package `PAM` (Partitioning Around Medoids) and `WGCNA` (Weighted Gene Coexpression Network Analysis).

Functional analysis

In the genes grouped by similar patterns we can identify over-represented (enriched) biological processes (`BP`). In our approach we conducted enrichment analyses applying hypergeometric tests (with p-value < 0.001) as implemented in the GOStats package. The universe is defined accrding to the Affymetrix platform selected, or, in case of multiple platforms in a single experiment design, the universe is defined as the common and unique genes among all Affymetrix platforms. The subset, geneset, is defined either by the set of diferentially expressed (DE) genes between a test and a control condition (control versus treatment design), or by the union of the DE genes selected among the pairwise comparisons among groups in all other single-factor experimental designs. Ontology information for the gene and universe sets is extracted from the Gene Ontology Consortium database `(GO)`.

Execute GeNNet!

After the configuration of all parameters, this last step executes the GeNNet workflow. This can take some time, users can check the execution progress by accessing the tab `Console`. After the execution, the results are placed the directory `Results`, including a complete graph database for visualization, making more questions and having insights about the data.

B – Access RStudio

The database takes some time to initialize, it will be accessible only a few seconds after starting the container. To access Rstudio, the user needs to open the service in `http://localhost:8787` (username: rstudio, password: rstudio) and enter both the username and password as `rstudio`.

The Figure 3 represents the workflow steps implemented in R.

The directory `Module-A` contains a set of R functions that correspond to the steps of analysis described in: A – A user-friendly Shiny-based web interface to the workflow experiment.

The users can modify the R scripts to customize the analyses. Furthermore, new scripts and libraries can be added through the RStudio interface. This gives an idea about the flexibility of the system defined as workflow management.

Results

During the workflow execution (in RStudio or Shiny) a set of results are written in the directory `Results` which ensures the re-use and persistence of data.

C – Access the Graph Database

The graph database is an intuitive way for connecting and visualizing relationships. In the GeNNet platform there is an initial database defined by interactions among genes from STRING-DB. During the execution of the GeNNet workflow by shiny or RStudio, new nodes and connections are formed and added to the database.

The database is based on a NoSQL paradigm. It was built based on Neo4j link. We chose this database because it was a natural way of representing the interaction among the nodes (genes) and the nodes derived from analysis. Furthermore, this database is free to use, multiplatform and easy to use and manipulate the data.

Although a NoSQL database has no fixed schema, we define an initial schema to help and guide the database. The graph structure is shown in Figure 2. We describe the nodes, relationships and properties associated with this model in detail below.

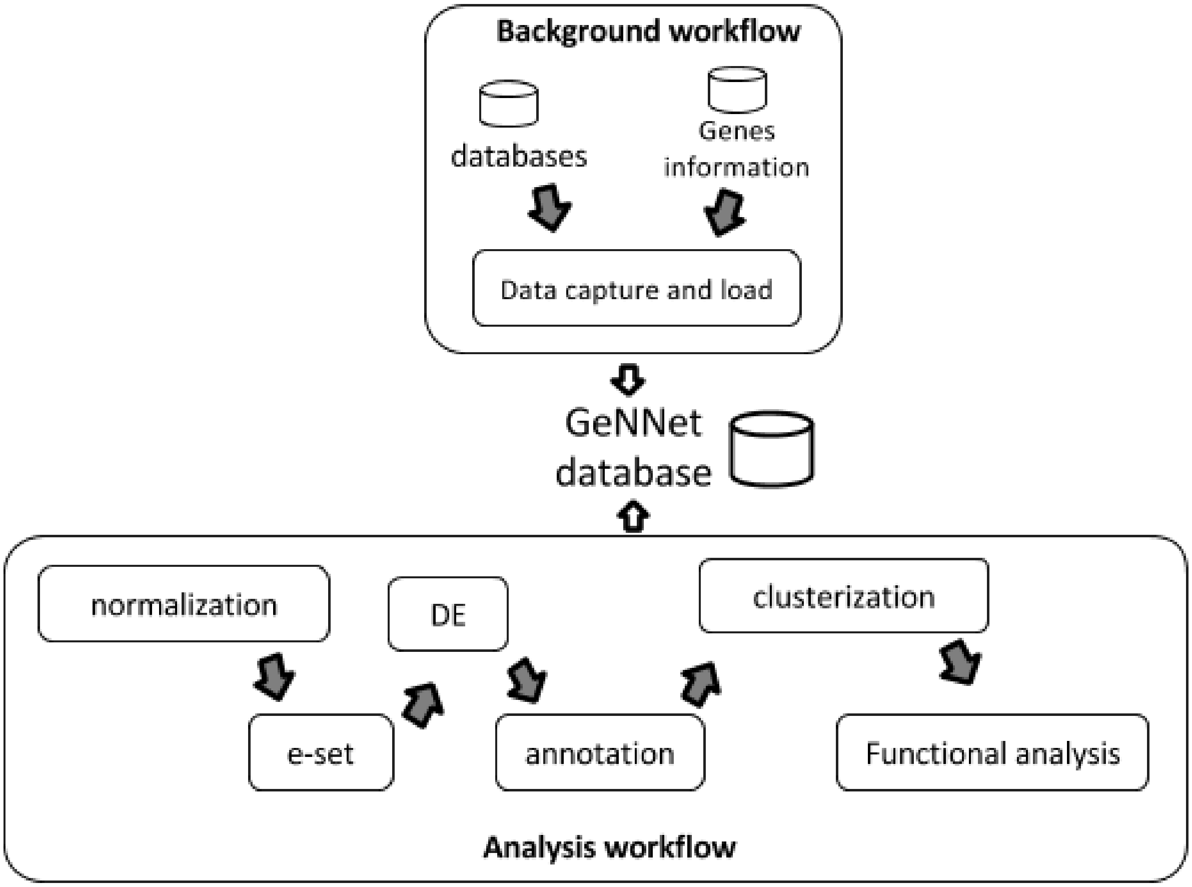

Figure 2: Figura 3: Workflow structure in GeNNet.

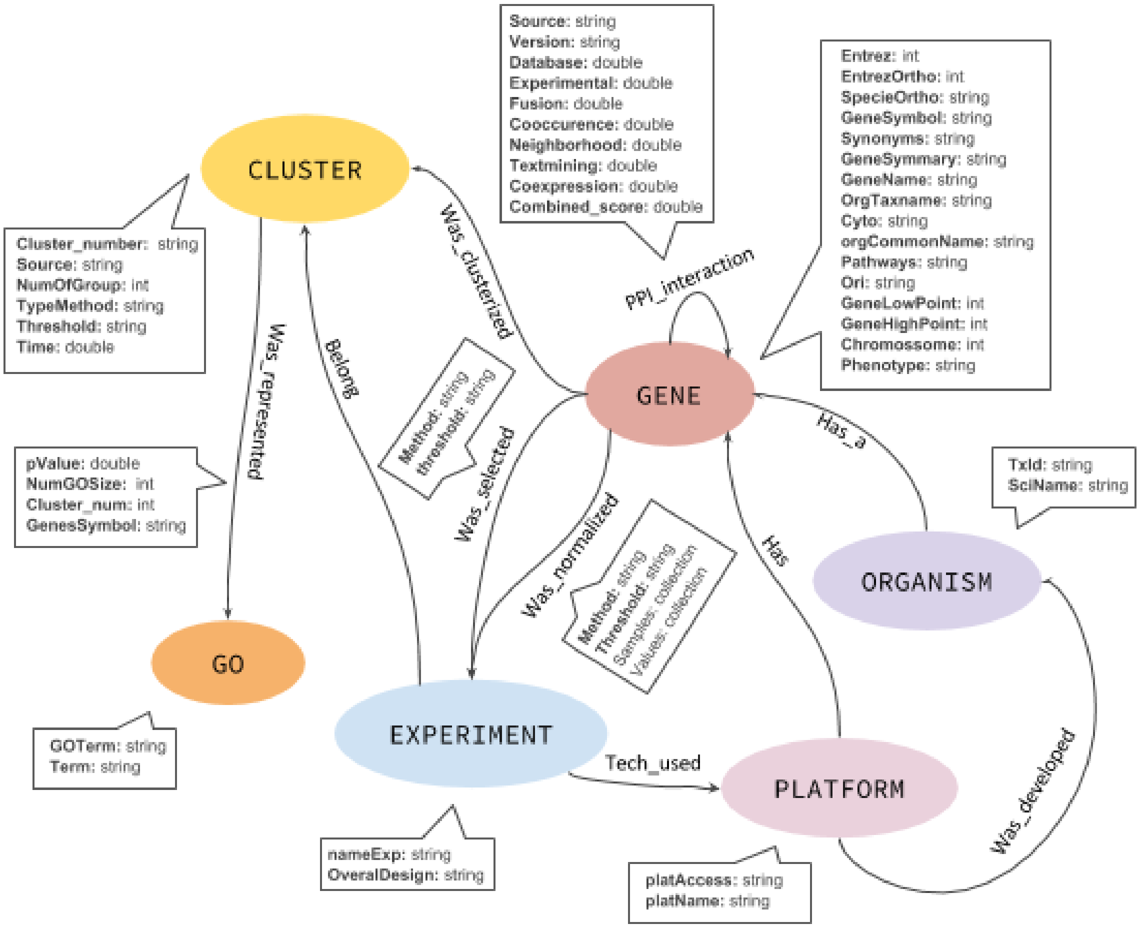

Figure 3: Figura 2: Graph database schema in GeNNet.

Genes

The nodes GENE represents the gene. The information was extracted from NCBI gene, which includes entrez_Id, gene symbol, summary, chromosome position, organism taxon, etc.

Organism

Describes the ORGANISM selected by the experiment. This node contains information including scientific name and Taxon id (TxId) from NCBI.

Experiment

The node EXPERIMENT contains information about the overal design, accession number from main transcriptome repositories (or other identifier).

Cluster

CLUSTER node is associated with a set of genes which were selected in biological function enrichment analysis. This node contains information about ...

GO

The GO node expresses the Gene Ontologies significantly associated with the cluster node.

Platform

The PLATFORM node expresses the Gene Ontologies significantly associated with the cluster node.

Has_a

This is a relationship among (ORGANISM)-[Has_a]->(GENE)
